# Right Ventricular Diastolic Dysfunction Before Coronary Artery Bypass Grafting: Impact on 5-Year Follow-Up Outcomes

**DOI:** 10.3390/jcm14041398

**Published:** 2025-02-19

**Authors:** Alexey N. Sumin, Anna V. Shcheglova, Nazeli D. Oganyan, Evgeniya Yu. Romanenko, Tatjana Yu. Sergeeva

**Affiliations:** 1 Federal State Budgetary Scientific Institution “Research Institute for Complex Issues of Cardiovascular Disease”, Blvd. Named Academician L.S. Barbarasha, 6, 650002 Kemerovo, Russia; nura.karpovitch@yandex.ru (A.V.S.); sergeeva.tatiana65@yandex.ru (T.Y.S.); 2 Federal State Budgetary Educational Institution of Higher Education “Kemerovo State Medical University” of the Ministry of Health of the Russian Federation, Voroshilova Str., 22 A, 650056 Kemerovo, Russia; oganyannazeli@mail.ru (N.D.O.); romanenkoe064@gmail.com (E.Y.R.)

**Keywords:** coronary artery bypass grafting, right ventricle, diastolic function, long-term postoperative results

## Abstract

**Background:** The aim of this study was to assess the effect of right ventricular diastolic dysfunction on the results of 5-year follow-up of patients after coronary artery bypass grafting (CABG). **Methods:** Patients were enrolled in this prospective observational study examined before planned CABG from 2017 to 2018. In addition to the baseline preoperative indicators and perioperative data, the initial parameters of the left and right ventricle (RV) systolic and diastolic function were assessed. The long-term results after CABG were assessed after 5 years. The following endpoints were recorded in the remote period: coronary and non-coronary death, non-fatal myocardial infarction (MI), repeat myocardial revascularization. **Results:** The results of long-term follow-up were assessed in 148 patients, during which time MACE was registered in 43 patients (29.1%). In the group with MACE before CABG, a history of myocardial infarction (*p* = 0.008), functional class 3 NYHA of chronic heart failure (CHF) (*p* = 0.013), an increase in the left ventricle size, a decrease in the e′/a′ ratio (*p* = 0.041), and the presence of the right ventricle diastolic dysfunction (*p* = 0.037) were more often detected. Kaplan–Meier analysis revealed a better long-term prognosis (MACE-free survival) in the group without RVDD compared to the group with RVDD (*p* = 0.026). **Conclusions:** In patients after coronary artery bypass grafting, the development of adverse events was associated with both clinical factors and the presence of right ventricular diastolic dysfunction. Survival analysis revealed a worse prognosis in patients with preoperative RVDD compared with patients without RVDD.

## 1. Introduction

The presence of chronic heart failure (CHF) worsens the prognosis during surgical interventions, both in cardiac and non-cardiac surgeries. Thus, the scales for assessing cardiac risk in non-cardiac surgeries include the presence of CHF as a risk-increasing factor, and the assessment of such a biomarker as NT-proBNP is proposed as an important step in assessing the preoperative condition of patients [1]. In cardiac surgeries, the EUROSCORE II scale proposes to assess the functional class of CHF and the left ventricular ejection fraction (EF) [2]. However, it has also been shown that other indices of intracardiac hemodynamics (except for left ventricular EF) can determine both immediate results and prognosis after cardiac surgeries [3,4,5,6,7]. Considering that, with systemic effects, the right heart chambers can be affected earlier than changes in the left heart chambers occur, the assessment of the right ventricle state in CHF [8,9] and before surgery has begun to attract the attention of researchers. As recent studies have shown, in non-cardiac surgeries, the presence of preoperative right ventricular dysfunction worsens the prognosis. For example, in elective abdominal surgeries, RV systolic dysfunction was independently associated with all-cause in-hospital mortality [10].

In cardiac surgeries, symptoms of heart failure are much more common, so there are significantly more publications on the perioperative assessment of right ventricular function. Basically, attention is paid to two aspects—the incidence of right ventricular failure in the postoperative period and the methods of its diagnosis and treatment [11,12,13,14], as well as the prognostic value of right ventricular dysfunction [15]. In the latter case, its systolic function is mainly studied [15,16,17], while only a few studies are devoted to diastolic dysfunction of the right ventricle [18,19,20]. At the same time, it is known that disorders of the diastolic function of the right ventricle occur earlier than disorders of its systolic function in experimental and clinical studies during the development of pulmonary hypertension [21,22,23,24]. In patients with chronic coronary syndrome, RV diastolic dysfunction is detected significantly more often than systolic dysfunction [25], and it is also associated with an unfavorable prognosis in cardiac surgeries [20,25]. It has previously been shown that the presence of RV diastolic dysfunction is associated with worsening not only in terms of the immediate results of CABG surgery but also during the 18-month follow-up after surgery [20]. However, it remains unclear whether the prognostic value of preoperative RV diastolic dysfunction is preserved during longer follow-up periods. This served as the rationale for conducting the present study, the aim of which was to assess the effect of right ventricular diastolic dysfunction on the results of 5-year follow-up of patients after coronary artery bypass grafting.

## 2. Material and Methods

### 2.1. Study Group Characteristics

Patients were enrolled in this prospective observational study from 2017 to 2018 at the Cardiology Department of the Federal State Budgetary Scientific Institution “Research Institute of Complex Issues of Cardiovascular Diseases” (Kemerovo). Patients with chronic coronary syndrome were examined before planned coronary artery bypass grafting (CABG). The study included patients with coronary artery disease with multivessel coronary artery disease, who were selected by a multidisciplinary team for direct myocardial revascularization. Before inclusion in the study, patients signed informed consent to participate in the study. The study design and protocol were approved by the local ethics committee of the institution (protocol No. 20170118). The sample was formed on a continuous basis, the inclusion criteria were stable coronary artery disease at the time of hospitalization and satisfactory visualization during echocardiography, the purpose of hospitalization was to perform planned CABG. Exclusion criteria: the presence of severe comorbidities in the patient that worsen the somatic status, ejection fraction <40%, patients with a recent acute coronary syndrome (less than 6 months), atrial fibrillation at inclusion, refusal (withdrawal of consent) to participate in the study.

### 2.2. Data Collection

All patients underwent standard preoperative examination upon admission: laboratory tests and instrumental examinations, including echocardiography. Information on demographic, anamnestic, and clinical parameters was obtained from the patients’ medical records. A multidisciplinary team made the choice of surgical treatment tactics. CABG was performed under cardiopulmonary bypass conditions using the generally accepted standard technique. All patients underwent complete myocardial revascularization. In the hospital, upon admission, drug therapy was prescribed in accordance with the current clinical guidelines for the management of patients with coronary artery disease [26]. Upon discharge, patients were prescribed optimal drug therapy and given recommendations on lifestyle changes in accordance with the recommendations.

### 2.3. Echocardiographic Examination

Echocardiography was performed using a Vivid S5 (GE) system using an extended protocol that was described in detail earlier [25]. The results were interpreted in accordance with the basic principles of international recommendations [27]. Left heart analysis included assessment of the left ventricle (LV) size and volume parameters, left atrium (LA) diameter, LV myocardial mass, and left ventricular ejection fraction (LVEF). Right heart analysis included assessment of the right atrium (RA) and right ventricle (RV) size, right ventricular wall thickness in diastole (RVWD), and tricuspid annular systolic motion from end diastole to end systole (TAPSE). Left ventricular diastolic function parameters were assessed using Doppler imaging (early and late LV transmitral filling rates and their ratio, LV isovolumetric relaxation time). To assess the diastolic function of the RV, we used the velocities of early (Et) and late transtricuspid filling of the RV (At) and their ratio (E t/At). We used tissue Doppler ultrasonography with the assessment of the movement of the valve rings to assess the systolic function of the ventricles—mitral for the LV and tricuspid for the RV. To assess diastole for the left and right ventricles, we measured the velocity of early and late diastolic movements of the mitral and tricuspid valve rings and their ratio (e’, a’, e’/a’, E/e’; e’t, a’t, e’t/a’t, Et/e’t). Additionally, we assessed the Tei index of the left and right ventricles. At Et/At ratio values within 0.8–2.1, the diastolic function of the right ventricle was considered normal. At Et/At ratio values <0.8 or >2.1 and/or Et/et’ ratio > 6, RV diastolic dysfunction was diagnosed [28].

### 2.4. Follow-Up

**Figure 1:**
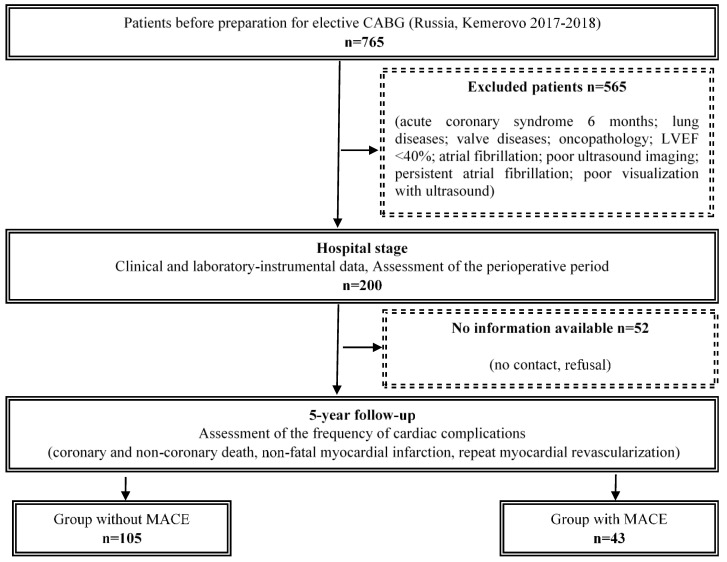
Flow chart of study design from screening to completion of trial. Notes: CABG—coronary artery bypass graft, LVEF—left ventricular ejection fraction, MACE—major adverse cardiac events.

**Figure 2:**
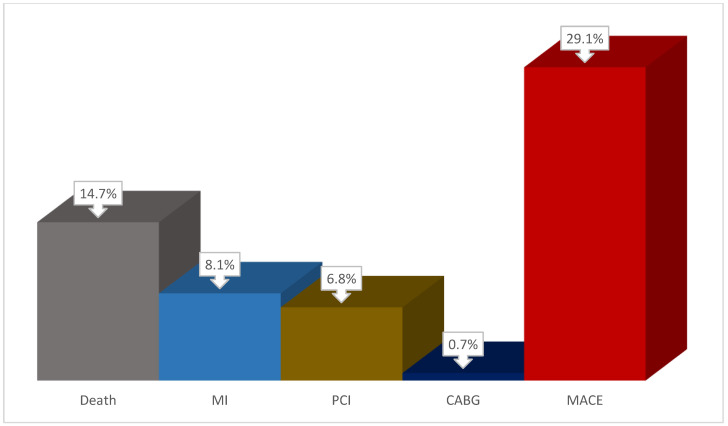
The structure of complications in follow-up after CABG. Notes: PCI—percutaneous coronary intervention, MI—myocardial infarction, CABG—coronary artery bypass graft, MACE—major adverse cardiac event.

The study design is presented in Figure 1. Long-term results of coronary artery surgery were assessed after 5 years. Information was collected by telephone (contact with the patient or his relative); data from local information systems and civil registration were also used. The following endpoints (MACE) were recorded in the remote period: coronary and non-coronary death, non-fatal myocardial infarction (MI), repeat myocardial revascularization (Figure 2). Based on the data obtained, groups with a favorable outcome (absence of cardiovascular events, n = 105) and with an unfavorable outcome (the presence of at least one of the events, n = 43) were formed.

### 2.5. Statistical Analyses

Statistical processing was performed using STATISTICA 8.0 (Dell Software, Inc., Round Rock, TX, USA) and SPSS 17.0 (IBM, Armonk, NY, USA). Normality of distribution for quantitative variables was assessed using the Kolmogorov–Smirnov test. Since the data distribution differed from normal, the results were presented as median and quartiles (25th and 75th percentiles). When comparing groups, the Mann–Whitney test was used for quantitative data and the χ^2^ (chi-square) test for qualitative indicators. To identify factors associated with the development of MACE during the observation period after CABG, a binary logistic regression (forward likelihood ratio) was used, including in the model indicators that differed significantly when comparing the groups. The performance of clinical and echocardiographic parameters in recognizing the risk of unfavorable prognosis after CABG was evaluated through receiver operating characteristic curve analysis. The level of critical significance (*p*) in the regression analysis was taken to be 0.05.

## 3. Results

During further follow-up, we were unable to obtain information on 43 patients who were lost to follow-up. We also received a refusal to continue participating in the study from 9 patients. As a result, we analyzed data on 148 patients (74.0% of the initial number of participants). The median follow-up of patients after CABG was 5.7 years. In this case, a fatal outcome was observed in 22 (14.7%) patients, myocardial infarction developed in 12 (8.1%) patients, PCI was performed in 10 (6.8%) patients, and CABG was performed in 1 (0.7%) patient. In total, MACE was detected in 43 (29.1%) patients (Figure 2).

The results of a comparison of groups with/without MACEs during long-term follow-up are presented in Table 1, Table 2, Table 3 and Table 4. The median age of the included patients was 64.0 (60.0; 69.0) years. The majority of patients were male in both groups. When analyzing the clinical and anamnestic data, the patient groups were homogeneous in all respects except for a history of MI. In the group with an unfavorable prognosis, a history of MI was observed significantly more often (in 76.7% of cases) than in the group with a favorable prognosis (in 53.3%, *p* = 0.008). In the group with the presence of MACEs, signs of functional class 3 chronic heart failure were more often detected (in 41.9% of cases versus 19.1%, *p* = 0.013). During hospitalization, there were no significant differences in the frequency of prescribing basic drugs (antiplatelet agents, beta-blockers, statins, ACE inhibitors, and CCBs). In the intergroup analysis of biological marker concentrations at the hospital stage, the median triglyceride (TG) was significantly lower in the group with an unfavorable prognosis (1.3 mmol/l) compared with the group with a favorable prognosis (1.5 mmol/L; *p* = 0.009). The overwhelming majority of patients (89.9%) had multivessel coronary lesions, according to coronary angiography data (Table 1). Perioperative characteristics are presented in Table 2. The groups were comparable in cardiopulmonary bypass (CBP) duration, aortic cross-clamp time, the number of shunts, and the volume of simultaneous intervention.

When analyzing the data of structural and functional indices of the left heart, it was found that the end-systolic dimension (ESD) and, accordingly, the left ventricular ESD index prevailed in patients with MACEs (*p* < 0.05). However, the median left ventricular ejection fraction was comparable in both groups and was within the normal values (≤60.0%). When studying the indices of diastolic function of the LV, a significant decrease in the ratio of the velocities of the early and late motions of the mitral valve annulus (e’/a’) was noted in patients with MACEs compared to the group without MACEs (*p* = 0.041) (Table 3).

When comparing the linear indices characterizing the size of the RV, no significant differences were found between the groups. The indices of the right ventricle systolic function (TAPSE and s’t) were comparable in the study groups. When evaluating transtricuspid flows in the group with an unfavorable outcome, a statistically insignificant decrease in the Et/e’t ratio was revealed. The RV myocardial function index (Tei index) also had no reliable differences (*p* = 0.335) between the studied groups. At the same time, the incidence of RV diastolic dysfunction in the group with an unfavorable prognosis was higher (56.1% versus 37.1%; *p* < 0.037) (Table 4).

**Figure 3:**
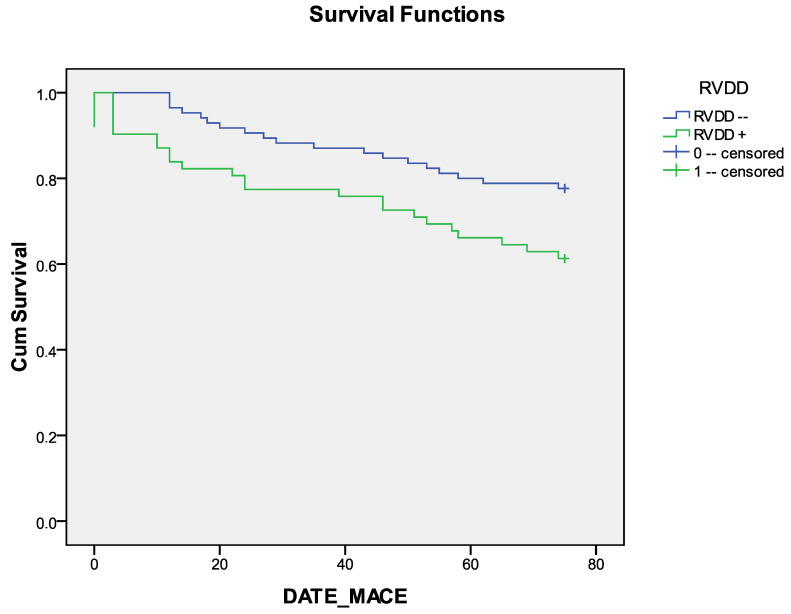
Long-term survival without MACEs after coronary artery bypass grafting in groups with/without RVDD. Notes: MACE—major adverse cardiovascular event, RVDD—right ventricular diastolic dysfunction.

The median survival time after CABG without MACEs was 65.7 +/− 2.2 in the group without RVDD and 57.1 =/− 3.5 in the RVDD group (Table 5). Kaplan–Meier curves revealed a better long-term prognosis (MACE-free survival) in the group without RVDD compared with the group with RVDD (Figure 3). The differences between the groups were statistically significant for the log-rank test, Breslow and Tarone–Ware tests (*p* = 0.026, *p* = 0.022 and *p* = 0.023, respectively, Table 6).

The results of binary logistic regression analysis showed that independent association with the development of MACEs in the long-term follow-up after CABG was determined only for clinical parameters (myocardial infarction in anamnesis and functional class 3 CHF NYHA), but not for echocardiography data (Suppl. Table S1). The logistic regression model was statistically significant, 2(2) = 13.229, *p* = 0.001. The model explained 12.4% (Nagelkerke R2 criterion) of the variance in the development of MACEs and correctly classified 73.8% of cases (Suppl. Tables S2–S4). The association of the initial parameters with the development of MACE is presented in Suppl. Figure S1. As shown in Suppl. Table S9, the areas under the curves were maximal for III functional class CHF NYHA (0.621) and myocardial infarction in anamnesis (0.614). However, these and the rest of the parameters (left ventricular EF and RVDD) were <0.7, indicating an unacceptable ability to distinguish.

## 4. Discussion

In this study, it was shown that a history of myocardial infarction, manifestations of FC III NYHA CHF, an increase in the size of the left ventricle, and the presence of diastolic dysfunction of the left and right ventricles was associated with an unfavorable long-term prognosis after CABG. In addition, Kaplan–Meier analysis revealed a better long-term prognosis (MACE-free survival) in the group without RVDD compared to the group with RVDD.

In the few previous studies, the main attention was paid to the effect of RV diastolic dysfunction on the immediate results of cardiac surgery. In patients with a decrease in LV ejection fraction (<35%), significantly reduced preoperative RV diastolic dysfunction (increased Et/Et’) and the absence of suitable target bypass vessels were independent risk factors for in-hospital mortality after CABG [18]. The authors conclude that in this cohort of patients, the assessment of preoperative RV diastolic dysfunction will be useful for predicting early death after CABG, and the Et/Et’ ratio ≥ 10 is significantly associated with early death after coronary artery bypass grafting [18]. At the same time, in patients without severe LV systolic dysfunction, it was not possible to identify the effect of RV diastolic dysfunction on in-hospital mortality [19,29]. Nevertheless, the adverse effect of RVDD on the immediate results of CABG surgery was still observed in these studies. Thus, an increase in the Et/E’t value (a marker of RV diastolic dysfunction) was associated with an increased risk of postoperative atrial fibrillation [19]. The presence of preoperative RVDD was associated with the development of postoperative heart failure after CABG [29].

To date, the impact of RV dysfunction on long-term prognosis after CABG has been poorly studied. Among patients with low LV ejection fraction during long-term follow-up (median follow-up—4.7 years) after CABG, only two indicators—RVEF and STS Risk Score—were independently associated with the development of both cardiovascular and overall mortality [30]. In a cohort of patients with preserved LV ejection fraction, it was shown that another indicator of RV function—right ventricle myocardial performance index—was independently associated with long-term mortality (with a follow-up of 3.5 years) after CABG (along with another indicator—the E/e’ ratio—reflecting the pressure in the left heart chambers) [16].

We have previously shown that the prognostic value of the presence of RVDD was noted one and a half years after CABG [20]. In the present study, we showed that even with a longer follow-up, the group without RVDD before CABG had significantly better long-term survival without MACEs than the group with RVDD. It should be noted that we obtained more accurate results in the analysis when analyzing the Kaplan–Meier curves than with binary logistic regression analysis, in which only clinical parameters had an independent effect on the prognosis, which was not observed for echocardiography parameters. This is due to the fact that when using logistic regression to assess time-dependent results, it is less accurate (since this analysis does not distinguish between the time to the event).

The clinical significance of this study is that it highlights the need to assess not only systolic but also diastolic functions of the right ventricle to predict long-term outcomes after CABG surgery. The issues of the most optimal markers of diastolic dysfunction (whether only Et/e’t and Et/At should be assessed [15] or whether the presence of RV diastolic dysfunction should be assessed in general [20]) remain unexplored. It is possible that persistent RV dysfunction observed one year after CABG surgery may provide additional prognostic information [31]. For example, in patients with CHF, a persistent decrease in RV systolic function over a year was associated with a subsequent unfavorable prognosis [9]. Other areas of research may include studying the prognostic value of other methods for assessing RV function, as discussed in a recent review, such as magnetic resonance imaging [32], studying intracardiac hemodynamics, and assessing the stiffness of the right sections, as well as right ventricular arterial coupling [15,33]. Currently, cardiovascular magnetic resonance (CMR) is used before CABG surgery both to assess myocardial viability [34,35] and to study the dynamics of right ventricular function [36,37]. Less information is available on the use of CMR in prognostic assessment in patients undergoing CABG with reduced LV systolic function [30]. However, the use of CMR requires additional expensive equipment, and the RV diastolic function indices we used are widely available in routine clinical practice. Additionally, there is a need to move from single-center studies to multicenter ones. In non-cardiac surgeries, such a step has already been taken—a prospective multicenter study IMPRoVE is planned to study perioperative RV dysfunction [38].

When evaluating the results of this study, it is necessary to take into account the existing limitations. Firstly, the study was conducted in one center, so its results cannot be generalized to other centers. Secondly, we did not evaluate the dynamics of echocardiography parameters in the postoperative period; it is quite possible that it could affect the prognosis during subsequent observation (apparently, this is the task of subsequent studies). Thirdly, we did not control drug therapy in the postoperative period (such therapy was carried out by outpatient physicians). Fourthly, this study did not include patients with comorbid conditions or valve lesions, so its results apply only to patients with isolated CABG. In addition, we tracked remote results in only 75% of patients; however, when conducting a separate analysis of preoperative parameters, the group of patients who dropped out of the study did not differ from those included in the analysis (Suppl. Tables S5–S8). Finally, in the present study, we assessed right ventricular function using only echocardiographic parameters without using intracardiac hemodynamic parameters. Recent data suggest that left ventricular myocardial strain parameters are useful for improving prognostic risk stratification in CABG surgery [39,40] in patients with suspected CAD [41], and right ventricular strain parameters are useful in cardiac surgery [42]. We used traditional indicators to assess the diastolic function of the right ventricle without taking into account additional methods for assessing this function (for example, we did not analyze the parameters of right ventricular deformation [43]). However, according to a multinational online survey, technologies such as global longitudinal strain and 3D echocardiography were rarely used to quantify RV function in clinical practice [44]. Therefore, in this study, we limited ourselves to using traditional RV function indices, which, in our opinion, will allow us to more widely apply its results in real practice. However, we considered it possible to present our data since we are the first to examine the impact of RV diastolic dysfunction on long-term CABG outcomes.

## 5. Conclusions

In patients after CABG, the development of adverse events (coronary and non-coronary death, non-fatal myocardial infarction (MI), and repeated myocardial revascularization) in the late period was associated with a history of myocardial infarction, with the presence of CHF III functional class, with an increase in the size of the left ventricle, a decrease in the e’/a’ ratio, and the presence of diastolic dysfunction of the right ventricle. Kaplan–Meier analysis revealed a better long-term prognosis (MACE-free survival) in the group without RVDD compared to the group with RVDD. In subsequent studies, it is necessary to clarify the possible prognostic impact on the prognosis of the right ventricle’s systolic and diastolic dysfunction if they are maintained in the postoperative period.

## Figures and Tables

**Table 1:** Preoperative demographic data and clinical measurements in groups with/without MACEs during follow-up after CABG.

Variable n (%), |Me (LQ;UQ)	Total (n = 148)	Group 1 Without MACE (n = 105)	Group 2 with MACE (n = 43)	*p*
	Demographic			
Sex, male	115 (77.7)	80 (76.2)	35 (81.4)	0.489
Age, years	64.0 [60.0;69.0]	64.0 [62.0;69.0]	64.0 [60.0;69.0]	0526
Body mass index, kgm^–2^	28.7 [25.8;31.2]	28.7 [26.2;31.1]	28.7 [24.6;31.2]	0.511
Smoking history active	48 (32.4)	34 (33.0)	14 (33.3)	0.971
Hypertension	140 (94.6)	102 (97.4)	38 (88.4)	0.032
Diabetes	37 (25.0)	27 (25.7)	10 (23.3)	0.753
Hyperlipidemia	95 (64.2)	70 (66.7)	25 (58.1)	0.325
	Cardiovascular history			
Angina	129 (87.2)	92 (87.6)	37 (86.1)	0.795
Myocardial infarction	89 (60.1)	56 (53.3)	33 (76.7)	0.008
Rhythm disturbances	29 (19.6)	19 (18.1)	10 (23.3)	0.472
Stroke	13 (8.8)	9 (8.6)	4 (9.3)	0.886
Previous PCI	25 (16.9)	19 (18.1)	6 (14.0)	0.541
CHF NYHA III FC	38 (25.7)	20 (19.1)	18 (41.9)	0.013
Carotid artery bilateral ≥50%	26 (17.6)	20 (19.1)	6 (14.0)	0.459
	Medical treatment			
β-blockers	138 (93.2)	98 (93.3)	40 (93.0)	0.946
Statins	143 (96.6)	103 (98.1)	40 (93.0)	0.121
CCB	109 (73.6)	78 (74.3)	31 (72.1)	0.783
ACEI	113 (76.4)	78 (74.3)	35 (81.4)	0.355
Aspirin	135 (91.2)	95 (90.5)	40 (93.0)	0.619
	Laboratory parameters			
Total cholesterol, mmol/L	4.4 [3.7;5.3]	4.6 [3.7;5.4]	4.2 [3.7;5.0]	0.296
LDL cholesterol, mmol/L	2.7 [2.1;3.5]	2.7 [2.1;3.6]	2.6 [1.9;3.3]	0.442
HDL cholesterol, mmol/L	1.13 [0.9;1.3]	1.1 [0.92;1.3]	1.16 [0.9;1.4]	0.583
Triglycerides, mmol/L	1.42 [1.1;2.2]	1.5 [1.2;2.2]	1.3 [1.0;1.8]	0.009
Glucose, mmol/L	5.7 [5.3;6.5]	5.7 [5.3;6.6]	5.7 [5.2;6.3]	0.471
Creatinine, mmol/L	85.5 [76.5;97.5]	85.0 [75.0;101.0]	86.0 [80.0;92.0]	0.958
NT-proBNP, pg/ml	67.4 [32.3;122.0]	62.0 [32.4;103.0]	78.5 [32.1;171.0]	0.465
	Coronary angiography			
1—coronary artery disease	12 (8.1)	10 (9.5)	2 (4.7)	0.324
2—coronary artery disease	61 (41.2)	42 (40.0)	19 (44.2)	0.639
3—coronary artery disease	72 (48.6)	50 (47.6)	22 (51.2)	0.695

Notes: MACE—major adverse cardiovascular events; CABG—coronary artery bypass surgery; PCI—percutaneous coronary intervention; CHF—chronic heart failure; NYHA—New York Heart Association; FC—functional class; ACEI—Angiotensin-converting-enzyme inhibitor; CCB—calcium channel blockers; NT-proBNP—N-terminal prohormone of brain natriuretic peptide.

**Table 2:** Perioperative parameters in groups with/without MACE during follow-up after CABG.

Variable n (%)|Me (LQ;UQ)	Total (n = 148)	Group 1 Without MACE (n = 105)	Group 2 with MACE (n = 43)	*p*
	Intraoperative characteristics			
Number of shunts	3.0 [2.0;3.0]	3.0 [2.0;3.0]	2.0 [2.0;3.0]	0.324
Cardiopulmonary bypass duration, minutes	77.0 [67.0;94.0]	77.0 [68.0;93.0]	75.5 [65.0;96.0]	0.711
Aortic cross-clamp time, min	51.0 [41.5;60.0]	50.5 [42.0;59.0]	52.0 [41.0;62.0]	0.636
Ventriculoplasty	11 (7.4)	6 (5.7)	5 (11.6)	0.213
Thrombectomy	7 (4.7)	4 (3.8)	3 (7.0)	0.409
Radiofrequency ablation	4 (2.7)	3 (2.9)	1 (2.3)	0.856
Carotid endarterectomy	18 (12.2)	13 (12.4)	5 (11.6)	0.898
Mitral valve replacement	1 (0.7)	0 (0)	1 (2.3)	0.116
Prosthetics of the aortic valve	1 (0.7)	1 (0.95)	0 (0)	0.521

Notes: MACE—major adverse cardiovascular event; CABG—coronary artery bypass surgery.

**Table 3:** Preoperative indicators of the left ventricle before surgery in groups with/without MACEs during follow-up after CABG.

Variable n (%), Me (LQ;UQ)	Total (n = 148)	Group 1 Without MACE (n = 105)	Group 2 MACE (n = 43)	*p*
	Structural indicators and systolic function			
Aorta, mm	3.55 [3.3;3.8]	3.6 [3.3;3.8]	3.5 [3.2;3.8]	0.864
LA, mm	4.5 [4.1;4.9]	4.5 [4.1;4.8]	4.6 [4.2;5.0]	0.256
EDD, mm	5.5 [5.3;6.1]	5.5 [5.2;6.1]	5.6 [5.4;6.1]	0.228
ESD, mm	3.6 [3.3;4.0]	3.6 [3.3;4.0]	3.7 [3.5;4.4]	0.02
ESDi, mm/m^2^	1.88 [1.72;2.1]	1.82 [1.7;2.0]	2.0 [1.8;2.3]	0.001
EDDi, mm/m^2^	2.91 [2.8;3.1]	2.9 [2.8;3.1]	3.1 [2.8;3.2]	0.06
EDV, mL	147.0 [132.5;187.0]	147.0 [130.0;187.0]	154.0 [141.0;187.0]	0.231
ESV, mL	51.0 [44.0;70.0]	51.0 [43.0;70.0]	51.0 [47.0;83.0]	0.151
ESVi, mL/m^2^	27.5 [21.8;35.1]	26.7 [21.6;34.3]	29.2 [25.3;39.8]	0.07
EDVi, mL/m^2^	79.3 [70.3;93.4]	77.3 [68.5;92.1]	83.6 [74.5;96.9]	0.08
LVEF, %	61.0 [56.0;65.0]	62.0 [56.0;66.0]	60.0 [52.0;63.0]	0.056
SV, mL	147.0 [132.5;187.0]	147.0 [130.0;187.0]	160.5 [135.0;180.0]	0.652
LVM, g	312.3 [259.0;374.6]	313.0 [242.1;374.0]	302.6 [284.0;375.0]	0.428
LVMi	149.7 [131.9;184.1]	146.6 [128.2;182.3]	163.0 [144.5;203.6]	0.112
IVST, cm	1.1 [1.0;1.2]	1.1 [1.0;1.3]	1.1 [1.0;1.2]	0.857
PW LV, cm	1.1 [1.0;1.2]	1.1 [1.0;1.2]	1.1 [1.0;1.2]	0.109
	Diastolic function			
IVRT, m/s	92.0 [90.0;98.0]	92.0 [90.0;98.0]	92.0 [90.0;98.0]	0.307
DT	236.0 [209.0;263.0]	229.0 [209.0;263.0]	236.0 [202.0;270.0]	0.489
E, cm/s	59.0 [46.0;66.0]	59.0 [46.0;67.5]	57.0 [44.0;65.0]	0.298
A, cm/s	67.5 [59.0;80.5]	67.0 [57.0;80.0]	70.0 [60.0;81.0]	0.475
E/A	0.77 [0.66;1.06]	0.79 [0.68;1.06]	0.75 [0.69;1.05]	0.237
e’, cm/s	9.1 [7.5;11.0]	9.4 [7.7;11.6]	8.5 [7.3;11.0]	0.093
a′, cm/s	9.9 [8.6;11.6]	9.8 [8.6;11.4]	10.5 [8.9;12.0]	0.323
e′/a′	0.87 [0.71;1.27]	0.91 [0.7;1.3]	0.76 [0.64;1.1]	0.041
s′, cm/s	9.0 [8.0;10.5]	9.0 [8.0;10.6]	9.0 [8.0;9.9]	0.714
E/e′,	6.2 [4.8;7.6]	6.2 [4.8;7.5]	6.5 [4.9;7.7]	0.652
Tei LV	0.32 [0.25;0.4]	0.33 [0.25;0.41]	0.28 [0.25;0.39]	0.317

Notes: MACE—major adverse cardiovascular event; CABG—coronary artery bypass surgery EDD—end-diastolic dimension; EDDi—end-diastolic dimension index; EDV—end-diastolic volume; EDVi—end-diastolic volume index; EF—ejection fraction; LA—left atrium; LVMi—left ventricular mass index; ESV—end-systolic volume; ESVi—end-systolic volume index; ESD—end-systolic dimension; ESDi—end-systolic dimension index; IVRT—isovolumic relaxation time; IVST—interventricular septum thickness; PW LV—posterior wall of the left ventricle; E—peak early diastolic left ventricular filling velocity; A—peak left ventricular filling velocity at atrial contraction; E/A—ratio of peak early diastolic filling velocity to peak filling velocity at atrial contract; e’—early diastolic mitral annular tissue velocity; a’—late diastolic mitral annular tissue velocity; e’/a’—ratio of the velocities of early and late movements of the mitral annulus; s’—systolic mitral annular tissue velocity; E/e’—ratio of the early diastolic velocity of mitral inflow to the early diastolic velocity of mitral annular motion; Tei LV—myocardial performance index left ventricular.

**Table 4:** Preoperative indicators of the right ventricle before surgery in groups with/without MACEs during follow-up after CABG.

Variable n (%), Me (LQ;UQ)	Total (n = 148)	Group 1 Without MACE (n = 105)	Group 2 MACE (n = 43)	*p*
	Structural indicators and systolic function			
RV, mm	2.0 [1.9;2.3]	2.0 [1.8;2.3]	2.0 [2.0;2.2]	0.902
RVth,MM [LQ, UQ]	0.4 [0.3;0.4]	0.4 [0.3;0.4]	0.4 [0.3;0.4]	0.962
TAPSE, mm	23.0 [21.0;26.0]	23.0 [21.0;26.0]	22.0 [21.0;26.0]	0.608
RVEF, %	55.0 [53.0;57.0]	55.0 [53.0;57.0]	55.0 [53.0;57.0]	0.713
RA, mm	40.0 [31.0;50.0]	40.0 [30.0;49.0]	39.0 [34.0;51.0]	0.988
mPAP, mmhg	12.0 [11.0;14.0]	11.0 [11.0;13.0]	13.0 [12.0;15.0]	0.137
sPAP, mmhg	27.0 [24.0;30.0]	27.0 [24.0;30.0]	25.0 [24.0;28.0]	0.755
	Diastolic function			
E_t_, cm/s	44.0 [37.0;49.0]	44.0 [38.0;48.0]	42.0 [35.0;49.0]	0.632
A_t_, cm/s	42.0 [34.0;49.0]	42.0 [34.0;48.0]	43.0 [35.0;49.0]	0.376
E_t_/A_t_	1.1 [0.8;1.4]	1.5 [1.3;1.7]	1.4 [0.99;1.5]	0.06
e′t, cm/s	9.4 [8.2;11.3]	9.4 [8.0;11.3]	9.4 [8.4;11.3]	0.933
a′t, cm/s	14.3 [12.1;16.0]	14.0 [12.0;15.6]	14.8 [12.9;17.0]	0.117
e′t/a′t, cm/s	0.69 [0.6;0.8]	0.7 [0.6;0.8]	0.68 [0.57;0.74]	0.233
s’_t_, cm/s	13.3 [11.9;15.0]	13.3 [11.9;15.0]	13.3 [11.9;15.2]	0.582
E_t_/e′t	4.4 [3.5;5.5]	4.5 [3.7;5.5]	4.3 [3.4;5.1]	0.577
RV Tei index	0.3 [0.23;0.37]	0.3 [0.24;0.37]	0.28 [0.22;0.36]	0.335
RVDD, n (%)	62 (41.9)	39 (37.1)	23 (56.1)	0.037

Notes: MACE—major adverse cardiovascular event; CABG—coronary artery bypass surgery RV—right ventricular; mPAP—mean pulmonary arterial pressure; sPAP—systolic pulmonary arterial pressure; TAPSE—tricuspid annular plane systolic excursion; Tei—myocardial performance index; RVth—thickness of right ventricular wall in diastole; EF—ejection fraction; RA—right atrium; Et—early transtricuspid diastolic filling; At—late transtricuspid diastolic filling; e’t—early diastolic tricuspid annular tissue velocity; a’t—late diastolic tricuspid annular tissue velocity; e’t/a’t—ratio of early diastolic tricuspid annular tissue velocity to the late diastolic tricuspid annular tissue velocity; s’t—systolic tricuspid annular tissue velocity; Et/e’t—ratio of early transtricuspid diastolic filling to the early diastolic tricuspid annular tissue velocity; RVDD—right ventricular diastolic dysfunction.

**Table 5:** Average values of time to MACEs after CABG in groups with/without RVDD.

RVDD	Mean			
			95% Confidence Interval	
	Estimate	Std. Error	Lower Bound	Upper Bound
RVDD−	65.729	2.161	61.495	69.964
RVDD+	57.081	3.471	50.278	63.884
Overall	62.082	1.957	58.247	65.916

Notes: MACE—major adverse cardiovascular event, CABG—coronary artery bypass surgery, RVDD—right ventricular diastolic dysfunction.

**Table 6:** Comparison of long-term survival after CABG without MACEs in groups with/without RVDD (log-rank test, Breslow, Tarone–Ware tests).

	Chi-Square	df	Sig.
Log Rank (Mantel-Cox)	4.976	1	0.026
Breslow (Generalized Wilcoxon)	5.277	1	0.022
Tarone-Ware	5.132	1	0.023

Notes: CABG—coronary artery bypass surgery, MACE—major adverse cardiovascular event, RVDD—right ventricular diastolic dysfunction.

## Data Availability

The datasets used and/or analyzed during the current study are available from the corresponding author upon reasonable request.

## Supplementary Materials

The following supporting information can be downloaded at: https://www.mdpi.com/article/10.3390/jcm14041398/s1. Table S1. Association of variables before surgery with MACE during follow-up after CABG (binary logistic regression analysis, forward likelihood ratio); Table S2. Association of variables before surgery with MACE during follow-up after CABG (binary logistic regression analysis, forward likelihood ratio): Omnibus Tests of Model Coefficients; Table S3. Association of variables before surgery with MACE during follow-up after CABG (binary logistic regression analysis, forward likelihood ratio): Model Summary; Table S4. Association of variables before surgery with MACE during follow-up after CABG (binary logistic regression analysis, forward likelihood ratio): Classification Table; Table S5. Preoperative demographic data and clinical measurements in patient in follow-up and patients lost to follow-up; Table S6. Perioperative outcomes in patient in follow-up and patients lost to follow-up; Table S7. Preoperative indicators of the left ventricle before surgery in patient in follow-up and patients lost to follow-up; Table S8. Preoperative Indicators of the right ventricle before surgery in patient in follow-up and patients lost to follow-up; Table S9. Receiver operating characteristic curve analysis. Performance of baseline parameters in discriminating MACE development in follow-up after CABG. Area under the curve; Figure S1. Receiver operating characteristic curve analysis. Performance of baseline parameters in discriminating MACE development in follow-up after CABG.
