# Supplementary material for: Right Ventricular Diastolic Dysfunction Before Coronary Artery Bypass Grafting: Impact on 5-Year Follow-Up Outcomes
Source: J Clin Med. 2025 Feb 19;14(4):1398. doi: 10.3390/jcm14041398 (PMC11856731; doi:10.3390/jcm14041398)
Supplement: Supplementary file 1 [file jcm-14-01398-s001.zip › jcm-3431032-supplementary.pdf]

Suppl. Table S1. Association of variables before surgery with MACE during follow-up after CABG (binary logistic regression analysis, forward likelihood ratio)

								95% C.I.for EXP(B)	
		B	Lower	Upper	df	Sig.	Exp(B)	Lower	Upper
Step 1	CHF FC III NYHA	1.257	0.414	9.216	1	0.002	3.514	1.561	7.912
	Constant	-1.196	0.224	28.569	1	0.000	0.302		
Step 2	MI in anamnesis	0.832	0.426	3.820	1	0.051	2.299	0.998	5.297
	CHF FC III NYHA	1.072	0.426	6.339	1	0.012	2.923	1.268	6.735
	Constant	-1.696	0.360	22.200	1	0.000	0.183		

Notes: MACE – major adverse cardiovascular events; CABG – coronary artery bypass surgery, CHF – chronic heart failure, FC – functional class, NYHA – New York Heart Association, MI – myocardial infarction

Suppl. Table S2. Association of variables before surgery with MACE during follow-up after CABG (binary logistic regression analysis, forward likelihood ratio):

Omnibus Tests of Model Coefficients

		Chi-square	df	Sig.
Step 1	Step	9.203	1	0.002
	Block	9.203	1	0.002
	Model	9.203	1	0.002
Step 2	Step	4.026	1	0.045
	Block	13.229	2	0.001
	Model	13.229	2	0.001

Suppl. Table S3. Association of variables before surgery with MACE during follow-up after CABG (binary logistic regression analysis, forward likelihood ratio):

Model Summary

Step	-2 Log likelihood	Cox & Snell R Square	Nagelkerke R Square
1	167.092	0.061	0.087
2	163.066	0.087	0.124

Suppl. Table S4. Association of variables before surgery with MACE during follow-up after CABG (binary logistic regression analysis, forward likelihood ratio):

Classification Table

	Observed		Predicted		
			MACE_1		
			0	1	Percentage Correct
Step 1	MACE	0	86	16	84.3
		1	26	17	39.5
		Overall Percentage			71.0
Step 2	MACE	0	91	11	89.2
		1	27	16	37.2
		Overall Percentage			73.8

Suppl. Table S5. Preoperative demographic data and clinical measurements in patient in follow-up and patients lost to follow-up

Variable<br>n (%) Me (LQ;UQ)	Patients lost to follow-up (n =52)	Patients in follow-up (n=148)	p
Demographic			
Gender Male	38(73.1)	115(77.7)	0.498
Age, years	63.0[60.0;66.0]	64.0[60.0;69.0]	0.365
Body mass index, kgm–2	27.8[26.0;30.0]	28.7[25.8;31.1]	0.529
Smoking history active	10(19.2)	48(32.4)	0.081
Hypertension	49(94.2)	140(94.6)	0.921
Diabetes	17(32.7)	37(25.0)	0.282
Hyperlipidemia	33(63.5)	95(64.2)	0.925
Cardiovascular history			
Angina	45(86.5)	129(87.2)	0.908
Myocardial infarction	37(71.5)	89(60.1)	0.157
Rhythm disturbances	7(13.7)	29(19.6)	0.347
Stroke	5(9.6)	13(8.8)	0.857
Previous PCI	10(19.2)	25(16.9)	0.702
Carotid artery bilateral ≥50%	12(23.1)	26(17.6)	0.384
Medical treatment			
β-blockers	46(88.5)	138(93.2)	0.274
Statins	48(92.3)	143(96.6)	0.197
CCB	35(67.3)	109(73.7)	0.381
ACEI	41(78.9)	113(76.4)	0.713
Aspirin	50(96.2)	135(91.2)	0.245
Laboratory parameters			
Total cholesterol, mmol/L	4.7[3.7;5.4]	4.4[3.7;5.3]	0.563
LDL cholesterol, mmol/L	2.81[1.92;3.5]	2.6[2.1;3.5]	0.914
HDL cholesterol, mmol/L	1.13[0.9;1.35]	1.1[0.9;1.3]	0.986
Triglycerides, mmol/L	1.5[1.2;2.1]	1.4[1.1;2.2]	0.809
Glucose, mmol/L	5.7[5.2;6.6]	5.7[5.3;6.5]	0.622
Creatinine, mmol/L	80.5[71.0;92.5]	85.5[76.5;97.5]	0.08
NT-proBNP, pg/ml	45.8[20.6;150.0]	67.4[32.2;122.0]	0.317
Coronary angiography			
1-coronary artery disease	8 (15.4)	12 (8.1)	0.132
2-coronary artery disease	19 (36.5)	61 (41.2)	0.554
3-coronary artery disease	24 (46.2)	72 (48.7)	0.756

Notes: PCI - percutaneous coronary intervention; ACEI - Angiotensin-converting-enzyme inhibitor. CCB - cal-cium channel blockers; NT-proBNP - N-terminal prohormone of brain natriuretic peptide.

Suppl. Table S6. Perioperative outcomes in patient in follow-up and patients lost to follow-up

Variables<br>n (%) / |Me (LQ;UQ)	Patients lost to follow-up (n =52)	Patients in follow-up (n=148)	p
Intraoperative characteristics			
Number of shunts	3.0 [2.0;3.0]	3.0 [2.0;3.0]	0.984
Cardiopulmonary bypass duration. minutes	78.0 [62.0;96.0]	77.0 [67.0;94.0]	0.821
Aortic cross-clamp time. ml	51.0 [40.0;63.0]	51.0 [41.5;60.0]	0.998
Ventriculoplasty	6 (11.5)	11 (7.4)	0.361
Thrombectomy	2(3.9)	7(4.7)	0.791
Radiofrequency ablation	2(3.9)	4(2.7)	0.678
Carotid endarterectomy	5(9.6)	18(12.2)	0.621
Mitral valve replacement	0(0)	1(0.7)	0.552
Prosthetics of the aortic valve	0(0)	1(0.7)	0.552

Suppl. Table S7. Preoperative indicators of the left ventricle before surgery in patient in follow-up and patients lost to follow-up

Variable<br>n (%)|Me (LQ;UQ)	Patients lost to follow-up (n =52)	Patients in follow-up (n=148)	p
Structural indicators and systolic function			
Aorta, mm	3.6[3.4;3.9]	3.6[3.3;3.8]	0.506
LA, mm	4.4[4.0;4.6]	4.5[4.1;4.9]	0.06
EDD, mm	5.8[5.3;6.2]	5.5[5.3;6.1]	0.241
ESD, mm	3.7[3.3;4.3]	3.6[3.3;4.0]	0.280
ESDi, mm/m2	2.0[1.8;2.2]	1.9[1.7;2.1]	0.116
EDDi, mm/m2	3.02[2.8;3.2]	2.9[2.7;3.1]	0.077
EDV, mL	167.0[135.0;194.0]	147.0[132.5;187.0]	0.256
ESV, mL	53.0[43.5;70.0]	51.0[44.0;70.0]	0.568
ESVi, mL/m2	28.6[22.9;37.8]	27.5[21.8;35.1]	0.332
EDVi, mL/m2	86.2[72.2;98.8]	79.3[70.3;93.4]	0.142
LVEF, %	60.0[50.0;65.5]	61.0[56.0;65.0]	0.472
SV, mL	167.0[135.0;194.0]	154.0[130.0;187.0]	0.433
LVM, g	302.9[250.4;347.1]	312.3[259.0;374.6]	0.671
LVMi	154.9[127.0;182.1]	149.7[131.9;184.1]	0.872
IVST, cm	1.0[1.0;1.2]	1.1[1.0;1.2]	0.104
PW LV, cm	1.0[1.0;1.2]	1.1[1.0;1.2]	0.118
Diastolic function			
IVRT, m/s	92,0[90,0;98,0]	92,0[90,0;98,0]	0,256
DT	216,0[196,0;263,0]	2336,0[209,0;263,0]	0,109
E, cm/s	54,0[44,0;67,0]	59,5[46,0;66,0]	0,472
A, cm/s	66,0[59,5;74,5]	67,5[59,0;80,5]	0,388
E/A	0,8[0,7;1,2]	0,77[0,66;1,05]	0,818
e’, cm/s	9,0[8,0;10,8]	9,1[7,5;11,0]	0,948
a’, cm/s	10,1[8,0;11,9]	9,9[8,6;11,6]	0,854
e'/a'	0,9[0,7;1,3]	0,9[0,7;1,3]	0,835
s’, cm/s	9,2[8,3;10,1]	9,0[8,0;10,5]	0,818
E/e',	5,7[4,8;7,2]	6,2[4,7;7,6]	0,431
Tei LV	0,36[0,26;0,48]	0,32[0,25;0,4]	0,086

Notes: EDD—end-diastolic dimension; EDDi—end-diastolic dimension index; EDV—end-diastolic volume; EDVi—end-diastolic volume index; EF—ejection fraction; LA—left atrium; LVMi—left ventricular mass index; ESV—end-systolic volume; ESVi—end-systolic volume index; ESD—end-systolic dimension; ESDi—end-systolic dimension index; IVRT—isovolumic relaxation time; IVST—interventricular septum thickness; PW LV—posterior wall of the left ventricle; E—peak early diastolic left ventricular filling velocity; A—peak left ventricular filling velocity at atrial contraction; E/A—ratio of peak early diastolic filling velocity to peak filling velocity at atrial contract; e’—early diastolic mitral annular tissue velocity; a’—late diastolic mitral annular tissue velocity; e’/a’—ratio of the velocities of early and late movements of the mitral annulus; s’—systolic mitral annular tissue velocity; E/e’—ratio of the early diastolic velocity of mitral inflow to the early diastolic velocity of mitral annular motion; Tei LV—myocardial performance index left ventricular.

Suppl. Table S8. Preoperative Indicators of the right ventricle before surgery in patient in follow-up and patients lost to follow-up

Variable<br>n (%)|Me (LQ;UQ)	Patients lost to follow-up (n =52)	Patients in follow-up (n=148)	p
Structural indicators and systolic function			
RV, mm	2.0[1.8;2.2]	2.0[1.9;2.3]	0.301
СПЖд,мм [LQ, UQ]	0.4[0.3;0.4]	0.4[0.3;0.4]	0.064
TAPSE, mm	24.0[21.0;26.0]	23.0[21.0;26.0]	0.983
RVEF, %	56.0[53.0;58.0]	55.0[53.0;57.0]	0.564
RA, mm	42.0[34.0;49.0]	40.0[31.0;50.0]	0.515
mPAP, mmhg	12.0[11.0;13.0]	12.0[11.0;14.0]	0.442
sPAP, mmhg	26.0[24.0;28.0]	27.0[24.0;30.0]	0.630
Diastolic function			
E_t, cm/s	46.0[38.0;51.5]	44.0[37.0;49.0]	0.189
A_t, cm/s	43.5[40.0;47.0]	42.0[34.0;49.0]	0.504
E_t/A_t	1.1[0.8;1.4]	1.1[0.8;1.4]	0.884
e'_t, cm/s	9.7[8.2;11.1]	9.4[8.2;11.3]	0.873
a'_t, cm/s	13.9[12.1;16.1]	14.3[12.1;16.0]	0.542
e'_t/a'_t, cm/s	0.68[0.6;0.8]	0.69[0.6;0.8]	0.993
s'_t, cm/s	12.6[11.6;14.8]	13.3[11.9;15.0]	0.494
E_t/e'_t	4.5[3.7;5.7]	4.4[3.5;5.5]	0.383
RV Tei index	0.31[0.22;0.41]	0.3[0.23;0.37]	0.408
RVDD, n (%)	23(44.2)	62(42.5)	0.825

Notes: RV—right ventricular; mPAP—mean pulmonary arterial pressure; sPAP—systolic pulmonary arterial pressure; TAPSE—tricuspid annular plane systolic excursion; Tei—myocardial performance index; RVth—thickness of right ventricular wall in diastole; EF—ejection fraction; RA—right atrium; Et—early transtricuspid diastolic filling; At—late transtricuspid diastolic filling; e't—early diastolic tricuspid annular tissue velocity; a't—late diastolic tricuspid annular tissue velocity; e't/a't—ratio of early diastolic tricuspid annular tissue velocity to the late diastolic tricuspid annular tissue velocity; s't—systolic tricuspid annular tissue velocity; Et/e't—ratio of early transtricuspid diastolic filling to the early diastolic tricuspid annular tissue velocity; RVDD—right ventricular diastolic dysfunction.

Suppl. Table S9. Receiver operating characteristic curve analysis. Performance of baseline parameters in discriminating MACE development in follow-up after CABG. Area under the curve.

Test Result Variable(s)				Asymptotic 95% Confidence Interval	
	Area	Std. Error	Asymptotic Sig.	Lower Bound	Upper Bound
MI in anamnesis	0.614	0.050	0.029	0.517	0.712
III functional class NYHA CHF	0.621	0.053	0.021	0.516	0.725
LVEF	0.403	0.051	0.065	0.304	0.502
RVDD	0.596	0.052	0.066	0.495	0.698

Notes: MACE – major adverse cardiovascular events; CABG – coronary artery bypass surgery, CHF – chronic heart failure, FC – functional class, NYHA – New York Heart Association, MI – myocardial infarction, LVEF – left ventricular ejection fraction, RVDD - right ventricular diastolic dysfunction.

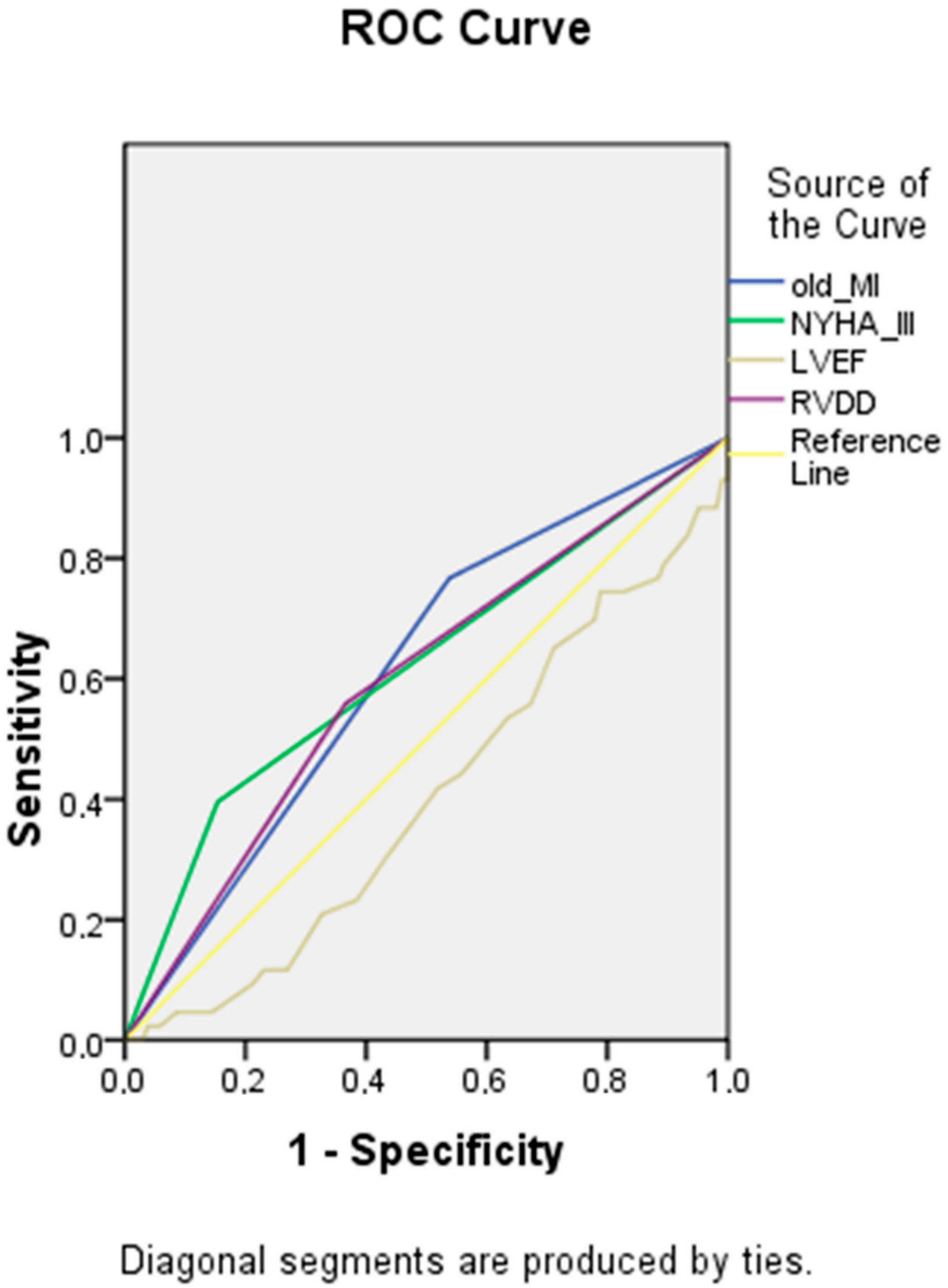

Suppl.

Figure S1. Receiver operating characteristic curve analysis. Performance of baseline parameters in discriminating MACE development in follow-up after CABG. Notes: MACE – major adverse cardiovascular events; CABG – coronary artery bypass surgery, NYHA - functional class of chronic heart failure, MI – myocardial infarction, LVEF – left ventricular ejection fraction, RVDD - right ventricular diastolic dysfunction.
